# Cartilage collagen damage in hip osteoarthritis similar to that seen in knee osteoarthritis; a case–control study of relationship between collagen, glycosaminoglycan and cartilage swelling

**DOI:** 10.1186/1471-2474-14-18

**Published:** 2013-01-09

**Authors:** Shahrzad Hosseininia, Lisbeth R Lindberg, Leif E Dahlberg

**Affiliations:** 1 Department of Orthopaedics, Lund University, Skåne University Hospital, Malmö SE-205 02, Sweden

**Keywords:** Cartilage, Hip, Osteoarthritis, Collagen, Glycosaminoglycan, Hydration

## Abstract

**Background:**

It remains to be shown whether OA shares molecular similarities between different joints in humans. This study provides evidence for similarities in cartilage molecular damage in osteoarthritic (OA) joints.

**Methods:**

Articular cartilage from osteoarthritic hip joints were analysed and compared to non-OA controls regarding collagen, glycosaminoglycan and water content. Femoral heads from 16 osteoarthritic (OA) and 20 reference patients were obtained from hip replacement surgery due to OA and femoral neck fracture, respectively. Cartilage histological changes were assessed by Mankin grading and denatured collagen type II immunostaining and cartilage was extracted by α-chymotrypsin. Hydroxyproline and Alcian blue binding assays were used to measure collagen and glycosaminoglycan (GAG) content, respectively.

**Results:**

Mankin and immunohistology scores were significantly higher in hip OA samples than in reference samples. Cartilage water content was 6% higher in OA samples than in references. 2.5 times more collagen was extracted from OA than from reference samples. There was a positive association between water content and percentage of extractable collagen pool (ECP) in both groups. The amounts of collagen per wet and dry weights did not differ statistically between OA and reference cartilage. % Extractable collagen was not related to collagen per dry weight in either group. However when collagen was expressed by wet weight there was a negative correlation between % extractable and collagen in OA cartilage. The amount of GAG per wet weight was similar in both groups but the amount of GAG per dry weight was higher in OA samples compared to reference samples, which suggests a capacity for GAG biosynthesis in hip OA cartilage. Neither of the studied parameters was related to age in either group.

**Conclusions:**

Increased collagen extractability and water content in human hip cartilage is associated with OA pathology and can be observed at early stages of the degenerative hip OA process. Our results suggest a common degradative pathway of collagen in articular cartilage of different joints. Furthermore, the study suggests that biochemical changes precede more overt OA changes and that chondrocytes may have a capability to compensate molecular loss in the early phase of OA.

## Background

Osteoarthritis (OA) of the knee and hip is a common cause of pain and reduced physical function in the elderly. Risk factors for OA differ across different joints. Whereas knee OA is associated with meniscectomy, obesity, muscle weakness and major injury, hip OA is commonly associated with congenital and developmental defects such as acetabular dysplasia, obesity and abnormal loading [1-5]. Felson et al. have suggested that OA in different joints may be discrete conditions but the final outcome is similar [2]. Although knee and hip OA risk factors suggest biomechanical etiopathogenesis [6], it still remains to be shown whether OA shares molecular similarities between different joints in humans. Despite an increasing prevalence of OA with age and that age is generally considered as a risk factor for OA [2], evidence for increasing incidence of OA with age is still conflicting [7].

The extracellular matrix of articular cartilage consists mainly of two macromolecules, type II collagen and the large aggregating proteoglycan, aggrecan. The collagen fibril is a heteropolymer primarily composed of collagen type II (more than 90%) with small amounts of type IX and type XI collagen [8]. Aggrecan, with its highly negatively charged glycosaminoglycans (GAGs), can bind up to 50 times its weight with water which creates water influx and a swelling pressure that is normally constrained by the tensile strength of the collagen fibrillar network [9].

In knee OA, disease progression is linked to matrix degradation and loss of molecules. Recent contrast-enhanced magnetic resonance imaging (MRI) studies support this assumption [10-12]. The impaired cartilage properties that follow degradation of matrix molecules may trigger subsequent damage to the collagen network and attempts at matrix repair [13]. Collagen degradation and loss may be particularly relevant, since network damage is generally considered as a point of no return regarding repair potential [14].

To evaluate the significance of molecular loss with respect to cartilage functional properties, it is necessary to estimate quantities of matrix molecules expressed in wet weight, as suggested by Maroudas et al. [9]. However, interpreting whether loss in molecular content is due to insufficient biosynthesis or dilution by oedema also necessitates dry weight analysis.

Hydroxyproline has been widely used to determine the presence and the metabolic activity of collagen in connective tissue [15-17]. α-Chymotrypsin enables digestion and thereby extraction of denatured collagen while sparing triple helical collagen [18,19]. Therefore, one can estimate the amount of extractable collagen, in part consisting of denatured type II collagen, in cartilage by combining these methods.

Previously, it has been shown that knee OA cartilage has an increased extractable collagen pool when treated by α-chymotrypsin, suggesting pathologic changes to the collagen network [16,18,20]. To further examine the generality of these observations, articular cartilage from osteoarthritic hip joints were analysed and compared to cartilage of well-defined hip fracture as non-OA controls.

## Methods

### Patients and cartilage

Femoral heads from 16 OA (10 women, 6 men, 45–81 years) and 20 reference patients (14 women, 6 men, 55–99 years) were obtained from hip replacement surgery due to OA or femoral neck fracture, respectively. Specimens were stored at −80°C until use. Ethics approval was obtained from the Research Ethics Committee, Lund University, Sweden and was in compliance with the Helsinki Declaration. Written informative consent for this study was obtained from participants.

OA cartilage was sampled from full depth regions with grossly intact surface, avoiding areas with macroscopic cartilage degeneration. Areas with evidence of osteophytes were also avoided. Reference cartilage was sampled from the superior weight-bearing part of the femoral head where cartilage was full depth and macroscopically intact.

Before dicing cartilage, it was soaked in 0.15 M NaCl at 4°C overnight to allow rehydration. Samples destined for collagen and GAG analyses were weighed wet, then freeze-dried and weighed dry to determine water content.

### Histology and immunohistochemistry

A cartilage block adjacent to the site sampled for biochemical analyses was examined for histology and immunohistochemistry. Sections 6 μm thick were cut at −20°C using a cryostat (Microm HM 560, Walldorf, Germany). One section was stained with Safranin O and Fast green and graded for histological changes as described by Mankin et al. (maximum grade was 13 due to exclusion of calcified cartilage from specimens) [21]. A second section was used for immunohistochemistry detecting denatured collagen II and graded as described by Hollander et al. [19].

### Chemical analyses of cartilage

Collagen: To remove the extractable collagen pool, 1 ml of 1 mg/ml α-chymotrypsin (TLCK Treated, type VII: from bovine pancreas, Sigma) in 50 mM Tris, pH 7.6, containing proteinase inhibitors (1 mM EDTA, 1 mM iodoacetamide and 10 μg/ml pepstatin-A) was added to 50 mg of diced cartilage sample. After incubation at 37°C overnight the supernatant was removed. The supernatant and the saline, used for equilibration, were diluted quantitatively 1:1 with 12 N HCl and the residue was immersed in 6 N HCl to be hydrolyzed at 110°C overnight. The hydrolysates were then dried at 90°C, reconstituted with 0.5 ml distilled water and dried again to remove traces of HCl. Finally, samples were dissolved in 0.5 ml distilled water and clarified by adding charcoal resin decolorizer (prepared from equal amounts of activated charcoal and AG-1 X8 anion exchange resin). The amount of hydroxyproline (μg/mg tissue) was measured by colorimetric methodology at 550 nm using L-4- hydroxyproline (Fluka) as a standard [22].

Results are reported as μg hydroxyproline, while in the discussion we refer to collagen content. The extractable collagen pool is expressed as percentage of the total collagen amount, as previously reported [23].

GAG: To another 10 mg of diced cartilage sample 1 μl of 19 mg/ml papain (papaya latex, EC 3.4.22.2, Sigma) in 0.1 M Tris–HCl, pH 7.2, containing 10 mM disodium EDTA and 5 mM cysteine-HCl, was added. After incubation at 60°C for 18 hours the digest was removed [24]. The amount of GAG was determined by a commercially available colorimetric kit using dye-precipitation of sulphated GAGs with Alcian blue [25].

### Statistical analysis

Differences between patient groups were analyzed by Mann–Whitney tests. Correlations were assessed using Spearman rank correlation coefficient. *P*-values less than or equal to 0.05 were considered significant.

## Results

Disease-related histological changes in OA cartilage were confirmed by Mankin and immunohistochemistry grading scores. The grades of OA and reference cartilage both in Mankin and immunostaining differed significantly, with Mankin grades of OA cartilage ranging from 2 to 6, and those from reference samples ranging from 0 to 3. There was no correlation between percentage of extractable collagen and Mankin or immunostaining grades in any of the groups (data not shown).

Hydroxyproline and GAGs were not detected in significant amounts in the saline solution used to soak cartilage over night (0.1-0.2% of total hydroxyproline and 1-2% of total GAG content).

Water content was almost 6% higher in OA cartilage than in reference cartilage (*P* ≤ 0.001) (Table 1). With respect to total collagen content, there was no difference between dry weights in OA and reference cartilage whereas wet weight comparison between the two groups showed a tendency to less collagen in OA samples due to increased water content (*P* = 0.083) (Table 1). In contrast, GAG content per dry weight was higher in OA than in reference cartilage (*P* = 0.012), whereas when measured by wet weight values were similar in OA and reference cartilage (*P* = 0.644) (Table 1).

**Table 1:** Age, histological and biochemical findings in patient groups

	**Reference (n = 20)**	**OA (n = 16)**	***P *value**
Age (years)	83 (77–85.5)	66.5 (61–75.5)	≤0.001
Mankin grade	2 (1–2)	4 (3.5-5)	≤0.001
Immunostaining score	0.5 (0.5-0.5)	2 (2–3)	≤0.001
ECP	0.45 (0.38-0.63)	1.04 ( 0.84-1.56)	≤0.001
% Hydration	68.1 (64–70.4)	73.6 (70.8-77.9)	≤0.001
GAG			
Wet	39.8 (34–45.6)	38.6 (34–45.2)	0.644
Dry	128.8 (119.6-135)	152.8 (129.4-165)	0.012
Total hydroxyproline			
Wet	25.3 (23.1-27.5)	22.4 (18–27.3)	0.083
Dry	79.6(72.2-87)	84.6 (76.4-95.7)	0.233

Reported values are medians (25–75 percentiles). Values of GAG and total hydroxyproline (i.e. total collagen) are expressed as micrograms per milligram wet and dry weight of cartilage tissue. ECP stands for % extractable collagen pool and is expressed as a percentage of the total amount of collagen.

**Figure 1:**
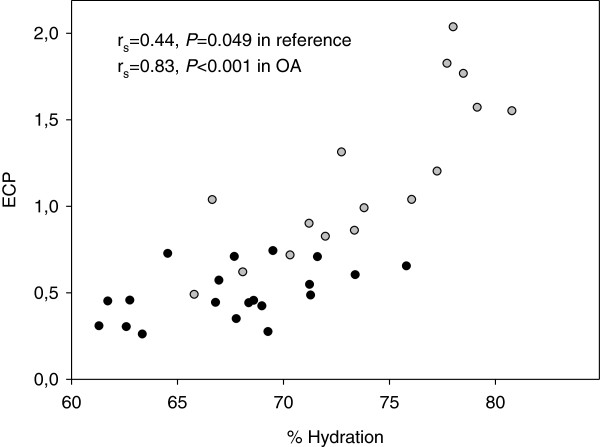
**The relationship between % hydration and extractable collagen pool.** Extractable collagen pool was expressed as a percentage of total amount of collagen. Open and closed circles represent OA and reference groups, respectively.

Overnight digestion with α-chymotrypsin extracted almost 2.5 times more collagen from OA than from reference samples (*P* ≤ 0.001) (Table 1). Water content was related to extractable collagen in OA (r_s_ =0.83, *P* < 0.001) and in reference cartilage (r_s_ =0.44, *P* = 0.049) (Figure 1). Water content was not related to the amount of GAG per dry weight in any of the two groups (r_s_ = 0.38, *P* = 0.146 in OA and r_s_ = −0.17, *P* = 0.468 in reference).

**Figure 2:**
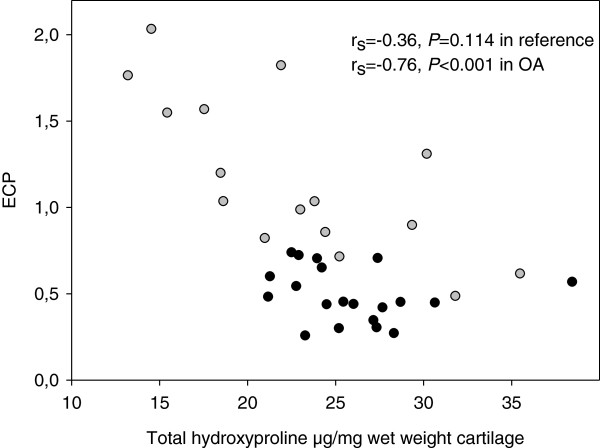
**The relationship between total collagen and extractable collagen pool.** Total collagen was measured as hydroxyproline per wet weight and extractable collagen pool was expressed as a percentage of total amount of collagen. Open and closed circles represent OA and reference groups, respectively.

Extractable collagen and total collagen per dry weight were unrelated in OA (r_s_ = −0.41, *P* = 0.112) and reference cartilage (r_s_ = 0.16, *P* = 0.484), but negatively related in OA cartilage when collagen was expressed by wet weight (r_s_ = −0.76, *P* < 0.001, Figure 2).

Neither extractable collagen, total collagen, GAG content (wet and dry weights) nor water content was related to age in OA or reference cartilage (data not shown).

## Discussion

Only full-thickness OA cartilage without any obvious macroscopic degenerative changes was included in this study to avoid comparison between full depth reference and partly damaged or thinner OA cartilage. As expected from macroscopic appearance, OA samples had a relatively low Mankin score. A Mankin grade assigned within the range 0–6 is accounted as “early phase of degenerative changes” [26]. However, in these samples cartilage oedema was present indicating that higher water content is evident at a very early stage of the disease. A strong relationship between cartilage oedema and collagen degradation has also been shown by Basser et al. [27]. That study suggests that the increased water content in OA cartilage is caused by loss of collagen network integrity, which in turn leads to decreased tensile stiffness and increased water imbibition, as proposed also by Maroudas et al. [9]. Experimental OA studies in rabbits (meniscectomy) and dogs (anterior cruciate ligament section model) have also shown increased cartilage swelling at early stages of OA [28,29]. The lack of relationship between water content and GAG content/dry weight in the present study confirms previous findings that swelling is not influenced by the amount of GAG [30].

Extractable collagen seems to be related to cartilage disease as evidenced by the positive relationship between extractable collagen and water content and that more collagen is extracted from OA than from reference cartilage (Figures 1, and 2). Reduced collagen content per wet weight in OA cartilage compared to non-OA cartilage, suggests inferior cartilage properties in OA (Figure 2) and points at the importance of protecting the collagen fibrillar network. An increased extractable collagen pool, which is inversely correlated to collagen content, has previously also been identified in knee OA cartilage [18,23]. Taken together, this suggests a common degradative pathway of collagen in articular cartilage of different joints. Studies showing similar findings in tendon and disc diseases support that this pathway is general in connective tissue [17,31].

A comparison of collagen content per dry and wet weights did not show differences between OA and reference groups (Table 1). This suggests either a loss of small amounts of collagen or a potential for chondrocytes to synthesis and deposit collagen in OA cartilage [32]. However, there is limited evidence that chondrocytes can recapitulate the overall collagen architecture if mature cartilage is damaged by injury or degeneration. In contrast, GAG content per dry weight was higher in OA samples than in reference samples (*P* = 0.012) whereas when measured by wet weight values were similar (*P* = 0.644). These differences draw attention to the importance of relating total molecular contents both to dry weight and to wet weight to provide maximum information on molecular content. This also suggests that cartilage may have a capacity to replace GAG; at least until advanced degradation occurs (our samples had an average Mankin score of 4). This repair capacity is known in the literature as hypertrophic repair [33-35] and has been visualized by radiography and by MRI [36,37]. Increments in GAG (mainly aggrecan) content have also been seen in studies of early cartilage damage [38]. Hypothetically, the lower amount of GAG in the relatively older reference group may to some extent be explained by decreased physical activity, as is suggested in a contrast-MRI study [39].

Several studies suggest age as a main risk factor for OA. Indeed, age is related to stiffer collagen, lower water content and malfunctioning chondrocytes in cartilage, all of which may predispose tissue damage [40-42]. However, overt cartilage loss is not a major feature of aging [43]. It is also apparent from experimental studies that matrix changes in OA cartilage are different from those in aged cartilage [44,45]. Albeit based on a limited number of cartilage samples, the present study suggests that OA matrix changes were disease-related rather than age-related. An alternative explanation to a cause-relationship between age and OA could be accumulation of micro damage due to exposure of joint load over a lifetime period.

There are some limitations to this study. Inherent in the use of hip fracture patients as controls, these were older than the patients who had OA (*P* ≤ 0.001) (Table 1). However, the lack of correlation between age and molecular OA changes in the present study supports the use of femoral neck fracture patients, regardless of age, as a control group in these types of studies. Regarding the validity of the reference group, it has previously been shown that cartilage from patients with femoral neck fracture is very similar to that of normal controls [46].

Cartilage was not sampled from the same position within the hip joint for all the samples. However, cartilage sampled from visibly intact full thickness regions from both groups make comparisons more valid. In support, Maroudas et al. have not found differences in GAG content in different locations of hip cartilage [47].

The hydroxyproline assay used to quantify collagen does not distinguish between different types of collagen, which may explain the lack of correlation between immunostaining of type II collagen and percentage extractable collagen in this study. We are in the process of conducting further studies to explore the origin of the collagen in the chymotrypsin extracted cartilage.

## Conclusions

Increased collagen extractability and water content in human hip cartilage is associated with OA pathology and can be observed at early stages of the degenerative OA process. Our results suggest a common degradative pathway of collagen in articular cartilage of different joints. Furthermore, the study suggests that biochemical changes precede more overt OA changes and that chondrocytes may have a capability to compensate molecular loss in the early phase of OA.

## Abbreviations

ECP: Percentage of extractable collagen pool; GAG: Glycosaminoglycan.

## Competing interests

Authors declare that they have no competing interest.

## Authors’ contributions

SH and LRL were involved in acquisition of data and drafting the manuscript. LRL assisted with immunohistochemistry. SH and LED were involved in analysis and interpretation of data. LED was involved in study design and revising the manuscript. All authors have read and approved of the final manuscript.

## Authors’ information

Shahrzad Hosseininia is an MD engaged in PhD study. Lisbeth R. Lindberg is Clinical Laboratory Scientist. Leif E. Dahlberg is MD, PhD and professor.

## Pre-publication history

The pre-publication history for this paper can be accessed here:

http://www.biomedcentral.com/1471-2474/14/18/prepub
